# Dual ectopic thyroid located at the tongue base and left parapharyngeal space with transient congenital hypothyroidism

**DOI:** 10.1210/jcemcr/luag252

**Published:** 2026-09-03

**Authors:** Kaho Kiuchi, Chieko Kusano, Naoaki Hori, Satoshi Narumi, Tomonobu Hasegawa

**Affiliations:** Department of Pediatrics, Ota Memorial Hospital, Gunma 373-8585, Japan; Department of Pediatrics, Keio University School of Medicine, Tokyo 160-8582, Japan; Department of Pediatrics, Ota Memorial Hospital, Gunma 373-8585, Japan; Department of Pediatrics, Ota Memorial Hospital, Gunma 373-8585, Japan; Department of Pediatrics, Keio University School of Medicine, Tokyo 160-8582, Japan; Department of Pediatrics, Keio University School of Medicine, Tokyo 160-8582, Japan; Department of Pediatrics, Keio University School of Medicine, Tokyo 160-8582, Japan; Department of Pediatrics, Kashiwa Tanaka Hospital, Chiba 277-0803, Japan

**Keywords:** congenital hypothyroidism, dual ectopic thyroid, transient

## Abstract

Ectopic thyroid is a rare developmental anomaly. In ectopic thyroid, thyroid tissue develops outside its normal position (anterior to the trachea), and it can occur within or outside the migratory pathway of the thyroid gland primordium. Ectopic thyroid tissue located in two sites is referred to as dual ectopic thyroid. This report describes a case of transient congenital hypothyroidism (CH) who had dual ectopic thyroid glands: one located at the base of the tongue (within the migratory pathway) and the other in the left parapharyngeal space (outside the migratory pathway). A 24-year-old woman, diagnosed with CH in the neonatal period, had been treated with levothyroxine. Since levothyroxine was discontinued at the age of 7 years, her thyroid function has remained normal. At 24 years of age, she was reevaluated because of throat discomfort. Although her thyroid function remained normal, ultrasonography revealed two ectopic thyroid masses. ^99m^Tc thyroid scintigraphy confirmed thyroid tissue in both locations. Genetic analysis did not reveal any suspicious genetic changes related to CH. Currently, she is under observation without treatment. This rare case of dual ectopic thyroid with transient CH underscores the importance of anatomical evaluation and long-term follow-up in patients with neonatal thyroid disorders.

## Introduction

Ectopic thyroid is a rare developmental abnormality wherein thyroid tissue emerges in a site other than the normal location anterior to the trachea because of abnormal migration of the gland [1]. Generally, it is located between the base of the tongue and the front of the trachea; this location is within the migratory pathway of the thyroid gland primordium. In other cases, it is located outside the thyroid primordium's migratory pathway, such as in the parapharyngeal space and heart [2, 3]. Ectopic thyroid glands located in two sites indicate dual ectopic thyroid [4]. In this condition, both thyroid tissues may be located in the primordium's migratory pathway, or one may be situated in the migratory pathway and another outside it. Congenital hypothyroidism (CH) caused by ectopic thyroid, including with dual ectopic foci, typically results in permanent CH because thyroid hormones cannot be sufficiently produced. To our knowledge, 30 studies on permanent CH caused by dual ectopic thyroid have been reported, including reviews, but none have reported transient CH [5]. In this case report, we present a case of transient CH with dual ectopic thyroid located at the base of the tongue (within the thyroid gland primordium's migration pathway) and at the left parapharyngeal space (outside the migration pathway). Since levothyroxine discontinuation at the age of 7 years, thyroid function has been normal.

## Case presentation

Our patient was a 24-year-old Japanese woman, who had no remarkable family history. Regarding medical history, she underwent a newborn mass screening test at 5 days old; at day 14, results revealed a high level of thyroid-stimulating hormone (TSH; 30.4 μIU/mL [30.4 mIU/L]; cutoff: 10 μIU/mL). On day 42, she was diagnosed with CH, with high levels of TSH (13.69 μIU/mL [13.69 mIU/L]; reference range for 2 months of age: 1.0-5.6 μIU/mL [1.0-5.6 mIU/L]), free triiodothyronine (FT3; 3.93 pg/mL [6.04 pmol/L]; reference range for 0-11 months of age: 1.8-4.8 pg/mL [2.8-7.2 pmol/L]), and free thyroxine (FT4; 0.93 ng/dL [12.0 pmol/L]; reference range for 0-11 months of age: 0.89-1.53 ng/dL [11.4-19.7 pmol/L]). No ultrasonographic or morphological evaluation was performed. Levothyroxine therapy was initiated. At the age of 7 years, having continuously been on levothyroxine (1.8 μg/kg/day), her thyroid function testing remained normal, with TSH (3.37 μIU/mL [3.37 mIU/L]) and FT4 (0.93 ng/dL [12.0 pmol/L]) within normal levels. With parents' consent, we discontinued levothyroxine. For the next 9 years, including during puberty, her thyroid function remained normal. At age 24, she visited our outpatient clinic for reevaluation of thyroid function because of an itchy and sore throat, which subsequently resolved spontaneously within a few months.

## Diagnostic assessment

**Figure 1:**
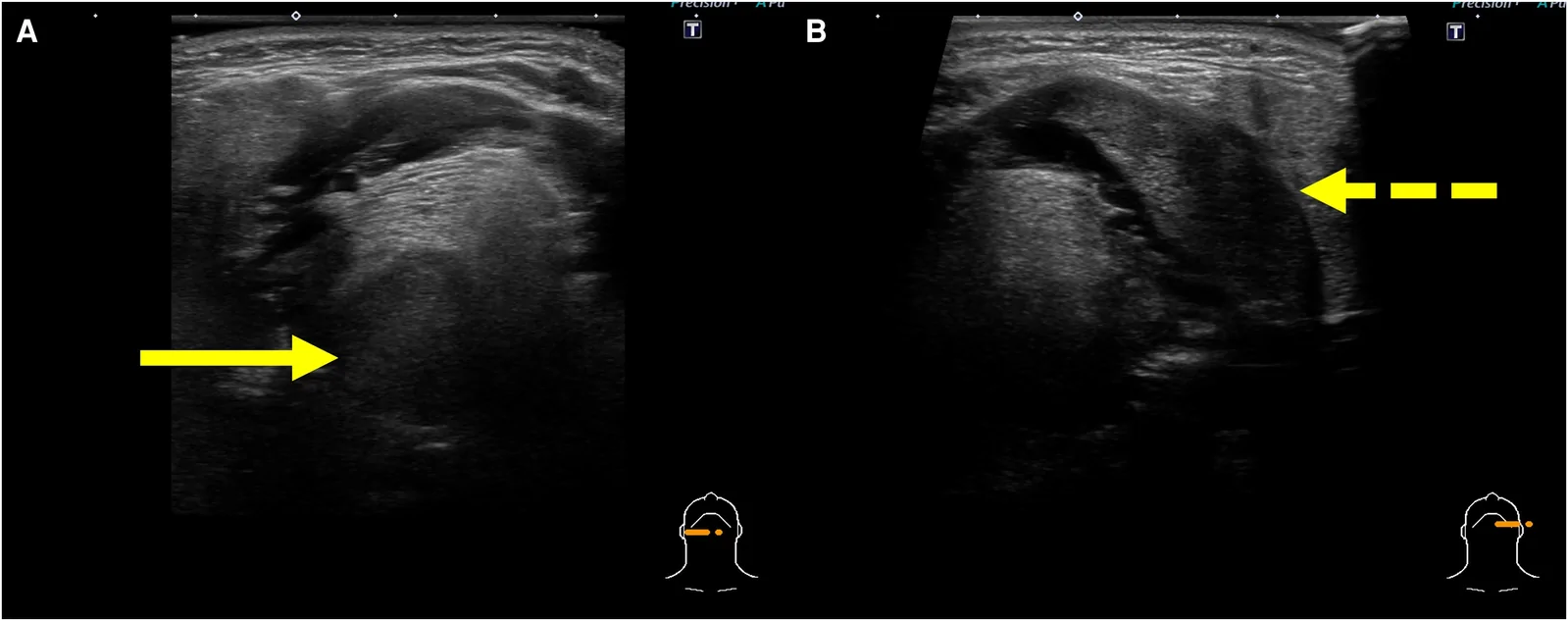
Images of thyroid ultrasonography. Two masses with clear demarcation and homogeneous internal composition. (A) At the base of the tongue. (B) At the left parapharyngeal space.

**Figure 2:**
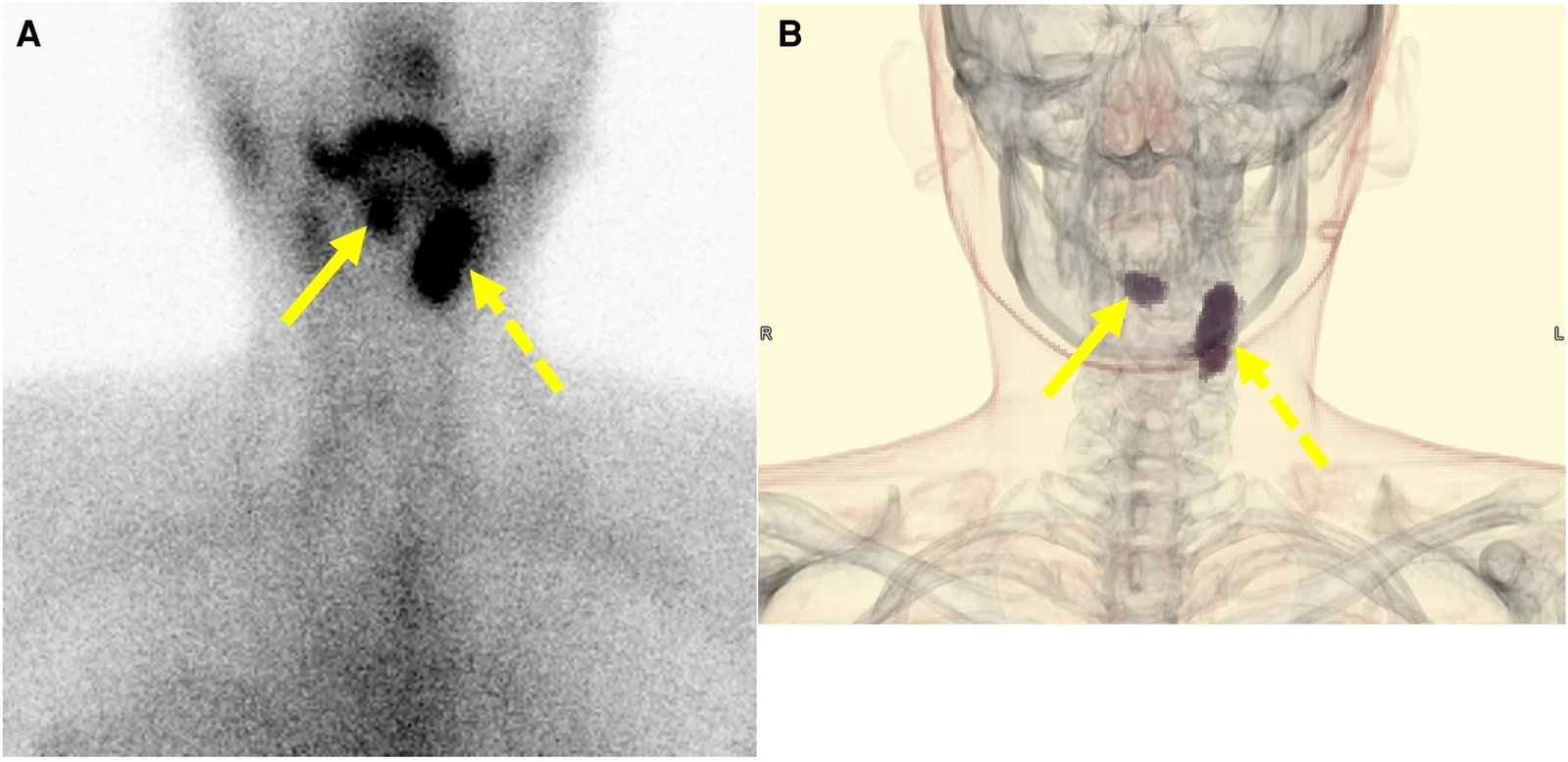
Images of ^99m^Tc thyroid scintigraphy and SPECT-CT. (A) Tracer accumulation is located at the base of the tongue and at the left parapharyngeal space. (B) 3D construction of SPECT-CT shows mass images at the same two locations. Abbreviations: SPECT-CT, single-photon emission computerized tomography with computed tomography.

She weighed 52.7 kg and was 159.1 cm tall. No oral or cervical masses were found on visual physical examination and palpation. Laboratory testing showed normal thyroid function (TSH: 1.77 μIU/mL [1.77 mIU/L]; FT3: 2.72 pg/mL [4.18 pmol/L]; FT4: 0.82 ng/dL [10.6 pmol/L]; thyroglobulin: 18.3 ng/mL [18.3 μg/L], reference range: 0-35.1 ng/mL [0-35.1 μg/L]). Ultrasonography failed to detect the orthotopic thyroid gland but detected two well-defined and internally homogeneous masses, with one at the base of the tongue (17.9 × 17.6 × 16.3 mm) and the other at the left parapharyngeal space (37.5 × 10.8 × 35.4 mm) (Fig. 1). Volumes were approximately 2.5 and 7.5 mL at the base of the tongue and the left parapharyngeal space, respectively. She has a dual ectopic thyroid in which the thyroid tissue is not located in its normal position. Although thyroid function baseline value was within the normal range, we deemed it necessary to evaluate thyroid function in detail and therefore performed a thyrotropin-releasing hormone stimulation test. The test revealed a normal TSH response (basal TSH: 1.86 μIU/mL [1.86 mIU/L]; peak value at 30 minutes: 18.8 μIU/mL [18.8 mIU/L]). Based on these results, it is considered that the ectopic thyroid tissue possesses normal thyroid function. ^99m^Tc scintigraphy showed tracer accumulation at both sites (Fig. 2), with intake rates of 0.15% and 0.42% at the tongue base and the left parapharyngeal space (reference range: 0.5-4.0%), respectively. Next-generation sequencing analysis of 19 CH-related genes (*DUOX2*, *DUOXA2*, *FOXE1*, *GLIS3*, *IGSF1*, *IYD*, *NKX2-1*, *PAX8*, *SECISBP2*, *SLC26A4*, *SLC26A7*, *SLC5A5*, *TG*, *THRA*, *TPO*, *TRH*, *TRHR*, *TSHB*, and *TSHR*) was performed. She did not have variants known to cause transient CH, including *DUOX2* and *DUOXA2* [6]. However, she had a heterozygous variant in *SLC26A4* (*c.2168A>G*, *p.His723Arg*; ClinVar Variation ID: 4825), the gene responsible for Pendred syndrome with autosomal recessive inheritance [7]. Furthermore, the rs9789446 allele, a risk allele for thyroid aplasia and ectopia (rs9789446 A allele), was homozygous [8].

## Treatment

Considering her euthyroid status, she has not been receiving any treatment.

## Outcome and follow-up

Taken together, the patient was diagnosed as having transient CH with dual ectopic thyroid gland. Presently, she is under observation without treatment. The symptom of throat discomfort resolved spontaneously and has not recurred. Although her current thyroid function is normal, she will be monitored regularly, given that pregnancy may worsen her thyroid function.

## Discussion

In the present case, the diagnosis of transient CH is definite for the following reasons: (1) the result of newborn screening for CH was positive; (2) her serum TSH level was high, whereas the FT4 and FT3 levels were normal on day 42; and (3) her thyroid function has been normal after levothyroxine discontinuation at the age of 7 years. Transient CH is highly likely caused by dual ectopic thyroid. Retrospectively, ectopic thyroid at the left parapharyngeal space could produce thyroid hormones to some extent, judging from tracer accumulation in ^99m^Tc scintigraphy. She did not have any known cause of transient CH other than dual ectopic thyroid. The most common genetic cause is biallelic variants of *DUOX2* [9, 10], but this was not found in the present case. *SLC26A4* (*c.2168A>G*, *p.His723Arg*) is “probably damaging” with a score of 1.0 (PolyPhen-2). Its pathogenicity is unknown. However, it has been reported that biallelic variants cause Pendred syndrome [11], and the patient is considered a carrier. The rs9789446 genotype is located on 2q33.3 and is unrelated to *SLC26A4.* The homozygous form of rs9789446 is present in 20.3% of the general population. It is associated with an increased risk of thyroid agenesis and ectopic thyroid [8]. The homozygous variants of rs9789446 A allele may increase a risk of dual ectopic thyroid. Other risk factors for transient CH, such as low birth weight, preterm birth, and maternal antithyroid medication use were not present [9, 10]. Iodine deficiency, iodine excess, and placental transfer of inhibitory TSH-receptor antibodies were unlikely according to her history.

The thyroid gland develops around the fourth week of gestation in the midline near the base of the tongue, descends along the thyroglossal duct to the anterior neck, and settles in its final position in front of the trachea around the seventh week of gestation. Although the mechanism is unclear, it is believed that ectopic thyroid tissue may implant outside the normal migration pathway due to abnormalities during this migration or during differentiation [4].

In 2016, Daniel-Tucker et al. showed that 15.7% of infants with ectopic thyroid had dual thyroid [5]. Dual ectopic thyroid reportedly has three clinical phenotypes. The first is permanent CH. In 2012, Wildi-Runge et al [12] reported that 9% of patients with permanent CH with ectopic thyroid have a dual ectopic thyroid, with an estimated incidence of 1 per 50 000–70 000 live births. The second is local symptoms, including foreign body sensation, dysphagia, and mass, without history of CH [2, 4]. In this category, some patients had mildly elevated TSH (subclinical hypothyroidism) [4]. The third is being asymptomatic. Dual ectopic thyroid glands could be discovered incidentally in adulthood [1, 13]. These three phenotypes should be considered continuous rather than distinct disease entities. We speculate that dual ectopic thyroid may cause transient CH, although such cases were previously undescribed [5].

Generally, the ectopic thyroid gland cannot secrete sufficient thyroid hormone. Indeed, all reported patients with CH due to dual ectopic thyroid gland prior to the present report had permanent hypothyroidism. When the physician confirms dual thyroid gland during replacement therapy, a trial to discontinue the treatment may be considered. In our patient, tracer accumulation was stronger or almost within the normal range in the left parapharyngeal space than in the base of the tongue by ^99m^Tc scintigraphy. The thyroid tissue in the left parapharyngeal space may have a function nearly equivalent to that of the orthotopic thyroid gland.

In our case, ^99m^Tc scintigraphy was useful for the diagnosis of dual ectopic thyroid. ^99m^Tc scintigraphy may be required when ultrasonography does not detect any thyroid gland at the orthotopic position but can also be considered when ultrasound detects a single ectopic thyroid focus. Dual ectopic thyroid foci may cause transient as well as permanent CH.

In summary, we report a case of transient CH associated with dual ectopic thyroid. The patient required levothyroxine treatment during early childhood but maintained normal thyroid function for 17 years after treatment discontinuation at age 7 years. Imaging at age 24 revealed ectopic thyroid tissue at the base of the tongue and the left parapharyngeal space, with the latter demonstrating nearly normal functional capacity on scintigraphy. This case expands the clinical spectrum of dual ectopic thyroid and highlights the importance of performing ^99m^Tc scintigraphy when ultrasonography fails to detect an orthotopic thyroid gland.

## Learning points

- Dual ectopic thyroid can have transient CH.
- In patients with CH with dual ectopic thyroid, a trial of discontinuing levothyroxine replacement therapy can be considered.
- When thyroid tissue cannot be identified at orthotopic position by ultrasonography, ^99m^Tc thyroid scintigraphy may detect a single or dual ectopic thyroid.

## Data Availability

Data sharing is not applicable to this article as no datasets were generated or analyzed during the current study.
